# A Perspective on Oral Immunotherapeutic Tools and Strategies for Autoimmune Disorders

**DOI:** 10.3390/vaccines11061031

**Published:** 2023-05-27

**Authors:** Akhilesh Kumar Shakya, Buddhadev Mallick, Kutty Selva Nandakumar

**Affiliations:** 1 Whitacre College of Engineering, Texas Tech University, Lubbock, TX 79409, USA; 2 Department of Zoology, Raniganj Girls College, Bardhaman 713358, West Bengal, India; 3 Department of Environmental and Biosciences, School of Business, Innovation, and Sustainability, Halmstad University, 301 18 Halmstad, Sweden

**Keywords:** autoimmune diseases, oral route, nanoparticles, antigen delivery system, transgenic plants, rheumatoid arthritis, type 1 diabetes

## Abstract

Oral immune tolerance is a physiological process to achieve tolerance against autoimmunity by oral ingestion of self-antigen(s) or other therapeutics. At the cellular level, oral tolerance suppresses autoimmune diseases by activating FoxP-positive and -negative regulatory T cells (Tregs) and/or causing clonal anergy or deletion of autoreactive T cells, affecting B cell tolerance. However, oral delivery of antigens/biologics is challenging due to their instability in the harsh environment of the gastrointestinal (GI) tract. Several antigen/drug delivery tools and approaches, including micro/nanoparticles and transgenic plant-based delivery systems, have been explored to demonstrate oral immune tolerance for different autoimmune diseases successfully. However, despite the effectiveness, variation in results, dose optimization, and undesirable immune system activation are the limitations of the oral approach to further advancement. From this perspective, the current review discusses the oral tolerance phenomenon, cellular mechanisms, antigen delivery tools and strategies, and its challenges.

## 1. Introduction

Autoimmune diseases, comprised of 80 different autoimmunity-dependent disorders, affect millions worldwide. Their prevalence is around 3–5% of the world population, with autoimmune type 1 diabetes being one of the most common diseases worldwide [1]. Rheumatic autoimmune diseases increase annually from 1% to 7.1% [2].

Autoimmune diseases are chronic, painful, and sometimes display life-threatening clinical manifestations that significantly affect the quality of life, causing enormous economic burdens to society. Despite intensive efforts at the preclinical and clinical levels, curing these diseases has yet to be feasible. However, new-generation biologics such as TNF-α inhibitors and B and T lymphocyte-targeted inhibitors (monoclonal antibodies and disease-modifying drugs) are currently available to tackle the autoimmune symptoms and effects temporarily. These biologicals considerably suppress chronic inflammation by blocking the synthesis/secretion of inflammatory cytokines or activating B and T cell receptors. However, the patients need potential drugs for treatment in milligram quantities at specific time intervals for a long duration, manifesting severe side effects [3]. Therefore, the antigen-specific immune tolerance (ASI) strategy can mitigate autoimmunity more effectively. Antigen-specific tolerance against autoimmunity is like the allergen-specific immunotherapeutic approach. The ASI concept was developed over a hundred years ago. Allergen-based immunotherapy is centered on the theory of repeatedly exposing the immune system to a specific allergen over a particular time duration. This results in the deviation from Th2-type to Th1-type responses and the induction of T regulatory cells (Tregs) to contain the unusual allergic immune reactions [4,5].

Similarly, antigen-specific immunotherapy targets autoimmune deviations by repeatedly exposing the immune system to autoantigen(s). In contrast to an allergic immune response biased towards Th2-type immune responses, an autoimmune response usually allied with Th1 and Th17 cell responses was developed against self-tissues [6]. Therefore, treating autoimmune disorders could be performed by suppressing the pathogenic Th1 and Th17 responses directed against the specific autoantigen(s). However, IL-21 cytokine produced mainly by T and natural killer T (NKT) cells can initiate an alternative pathway to induce the proinflammatory Th17 cells [7]. Repeated exposure of the immune system to antigen-specific immune tolerance aims to modulate the immune system for promoting tolerance against self-antigens. Over the past several decades, different antigen-specific or nonspecific immune tolerance strategies have been developed to alleviate the symptoms of autoimmune diseases. Herein, we presented oral immune tolerance mechanisms, antigen delivery methods and tools, and challenges associated with these oral immune tolerance strategies.

## 2. Oral Immune Tolerance

Immunological tolerance is the healthy state of the immune system discriminating self- and non-self-antigens, thereby protecting the host from external threats, for example, microbes. Failure of self-tolerance leads to the activation of autoimmune responses. Both central and peripheral tolerance mechanisms activate the regulation of self-reactive T cells, which will also affect B cell tolerance outcomes. For example, in an experimental autoimmune disease (Myasthenia gravis), IL-17-producing CD4^+^ T cells were shown to contribute to defects in B cell tolerance [8]. For a detailed review of the mechanisms of B cell tolerance involving clonal deletion, apoptosis, and receptor editing, refer to Nemazee (2017) [9]. Generally, in a healthy state, these mechanisms maintain immune homeostasis and inhibit initiating autoimmune responses. At the time of the breakdown of these tolerance mechanisms, self-reactive immune cells are activated and destroy the target tissues [10]. An aberrant activation of methylation pattern of differentially expressed genes and alteration cellular pathways in the immune cells (B and T cells and monocytes) of autoimmune diseases, \ [11], allows the development of antigen-specific or nonspecific immunotherapeutic strategies, including appropriate epigenetic alterations to restore the immune homeostasis.

**Figure 1:**
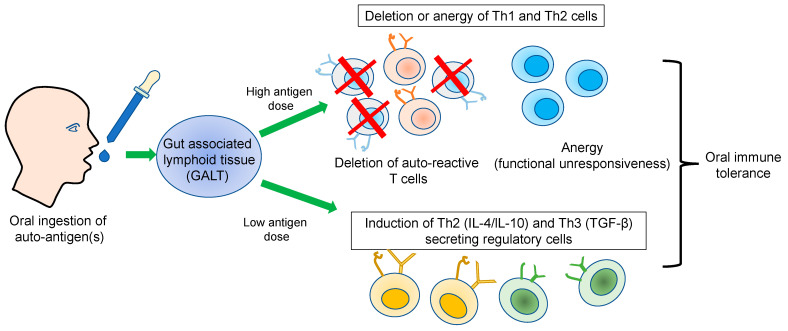
Schematic representation of the possible mechanism of oral immune tolerance against autoimmune disorders. The autoantigen(s) delivered through the oral route activates the gut-associated lymphoid system in a dose-dependent manner. A high antigen dose favors the deletion of autoreactive T cells or the anergy state. However, a low antigen dose activates the T cell regulatory response to suppress the autoimmune response.

Antigen-specific immune tolerance can be directed towards effector T cells through various mechanisms, including clonal deletion, anergy, or Treg activation. These promote the secretion of several anti-inflammatory cytokines to regulate the autoreactive T cells [12,13] (Figure 1). Anergy indicates an unresponsive status of T cells against an appropriate antigen/peptide. At first, several labs performed anergy experiments in vitro. For example, a high dose of peptide epitopes generated unresponsive T-cell clones against the same peptide [14]. Finally, however, compelling evidence of anergy was provided using an in vivo model, in which tolerance was achieved against autoimmunity by repeated exposure to the antigen and used to develop T cell receptor (TCR) transgenic mice.

Interestingly, a decline in the number of T cell clones was negligible after intravenous administration of the same peptide [15]. Similarly, the contribution of clonal deletion to antigen-specific tolerance was verified using TCR transgenic mice [16]. Deletion of peptide-specific CD4^+^T cells from the lymphoid organs after peptide exposure was dependent on apoptosis and/or Fas/Fas ligand-mediated cellular death pathways [17,18].

The Forkhead box protein 3-positive (Foxp3^+^CD25^+^CD4^+^) Tregs are essential for maintaining the self-tolerance and homeostasis of immune system [19]. Human Tregs consist of distinct subpopulations: CD45RA^+^FoxP3^lo^ resting Tregs, CD45RA^−^FoxP3^hi^ activated Tregs, which were suppressive under in vitro condition, and cytokine-secreting CD45RA^−^FoxP3^lo^ non-suppressive T cells [20]. By activating and expanding antigen-specific Tregs, they can be used to treat autoimmune diseases [21]. However, Tregs did not wholly prevent T1D, possibly due to their development and function/inhibition involving IL-6, plasticity, possible autoimmune T cell-mediated resistance to their functions, and associated cytokines (IL-10, IL-35), and the transforming growth factor beta (TGF-β), in promoting their activity and synthesis [22]. The absence of IL-7Rα expression could affect Treg numbers/functions [23,24]. Autoantigens stimulating autoreactive T cells may also activate natural Tregs. These cells could promote dominant self-tolerance; however, aberrations in the genetic and environmental factors affecting Tregs might cause autoimmunity [25]. Tregs-mediated suppression involves several steps, and a defect or promotion of each step can alter Tregs-mediated suppression’s effectiveness [26]. Besides producing a potent anti-tumor immune response, CTLA-4 deficiency in Tregs leads to the spontaneous development of systemic lymphoproliferation, lethal T cell-mediated autoimmunity disease, and a significantly increased production of IgE in mice [27]. Along with Tregs, activation of Th2-type, but suppression of Th1/17-type pathways can also contribute to the successful development of the immune tolerance [28]. It is well-known that TGF-β induces the Th17 development [29] and differentiation [30]. TGF-β regulates T cell proliferation by inhibiting the IL-2 production [31]. Several preclinical studies have shown that a deficiency in TGF-β leads to impaired immune tolerance and developing risks of autoimmune disorders in mice models [32,33]. For instance, Tregs highly expressed the TGF-β during oral antigen delivery to show the preventive effect in developing EAE [32]. Moreover, in the liver tissue of the EAE model, antigen delivery activated the Tregs in a TGF-β dose-dependent manner to achieve an autoimmune tolerance [34,35].

## 3. Immunological Basis of Oral Tolerance

The small intestine plays a vital role in achieving oral immune tolerance. Anatomically, the small intestine contains a villi structure lined by an epithelial layer and further covered by a thick mucus layer. The epithelial membrane appears as a selectively permeable membrane that allows nutrients and other fluids while blocking the entry of microbial agents or other intraluminal toxins. The epithelial layer maintains its discernment due to tight junctions between the cells that tightly close the intracellular space [36]. A few cells in the epithelial lining layer are immune-receptive cells with different membrane-bound toll-like receptors (TLRs) on their surface. These cells act as sensors to perceive upcoming microbial threats by activating toll-like receptors (TLRs) by binding to the intra-luminal toxins or other microbial products. Signaling through TLRs activates the recruitment of specific intracellular adaptor proteins to initiate the signaling cascades, culminating in the activation of NF-κB, MAP kinases, and interferon regulatory factors (IRFs). These transcription factors control the transcription of the genes encoding proinflammatory cytokines such as type I interferon, which is crucial for eliminating potential threats to the host [37]. Gottschalk et al. have identified different activation threshold levels of NF-κB and MAPK that could provide sequential barriers to producing inflammatory mediators [38]. Other cells of the epithelial layer are the microfold (M) cells, which are absorptive and reside on the top of lymphoid tissue payer patches [39]. M cells take foreign molecules nonspecifically and transport them across the epithelium to present to the underlying IgA-secreting B cells within the vault of the Peyer patch [40]. Alternatively, dendritic cells (DCs) can interact directly with the antigens by extending their dendrites’ [41]. Apart from the DCs, other immune cells, such as macrophages, B cells, and T cells, also present antigens to activate the immune system. Activated B and T cells leave the mucosal sites and migrate to the other lymph tissues to activate the immune system on other mucosal sites. Secreted cytokines and chemokines in the local environment control the expression of homing receptors (integrins) and mucosal tissue receptors (Addressins) of lymphocytes at the mucosal surface to control their migration to other mucosal sites [42].

### 3.1. Dose Effect

Oral immune tolerance is a complex phenomenon mediated by several factors, for example the dose and frequency of the administrated antigen(s). A low amount of antigen induces the activation of Tregs. At the same time, a high dose favors the induction of clonal anergy or the deletion of T cells to suppress the autoimmunity [43,44]. For instance, Ova-specific TCR transgenic mice fed with a high dose of Ova suppressed the autoimmune responses by deleting Ova-specific autoreactive T cells in the spleen and gut-associated lymphoid tissues (GALT). However, the mechanism of T cell deletion is unclear, though the activation of CTLA-4 might play an essential role in deleting autoreactive T cells [45]. In contrast to a low dose, a high dose of antigen activates the Tregs and leads to the secretion of TGF-β and IL-10, suppressing the autoimmune-mediated inflammatory responses. The activated Tregs then migrate to the lymphoid organs and inhibit the recruitment of effector cells at the target tissue. For example, Tregs’ activation in the experimental autoimmune encephalomyelitis (EAE) rat model was verified by feeding a low dose of myelin-binding protein (MBP) [46]. Generally, a low or high antigen dose effect on oral tolerance depends on the B7.1 and B7.2 molecules binding to CTLA-4 in T cells [47]. Similarly, oral immunotherapy of idiopathic pulmonary fibrosis (IPF) patients had a dose effect. Most patients with IPF have elevated circulating antibody levels against type V collagen. In a phase 1 trial, 30 antibody positive IPF patients showed improved lung symptoms after administering IW001 (type V collagen). The effect of a low oral dose of IW001 was comparable to the placebo group. However, a high amount of IW001 showed a positive therapeutic impact on the forced vital lung capacity and reduced C1q binding to anti-col(v) antibodies [48].

### 3.2. Bystander Effect

The bystander effect refers to suppressing autoreactive immune cells by ingesting a specific antigen that can stop the immune responses generated by another antigen. Generally, fed antigens structurally mimic another antigen, yielding this effect [49]. Under in vitro conditions, the bystander effect was demonstrated in a study where the splenic cells isolated from the mice fed with MBP antigen suppressed the proliferation of the Ova-specific T cells. On the other hand, spleen cells from Ova-fed animals showed a suppression of MBP-specific T cells [49]. The responsible factor for the suppression was TGF-β. Under an in vivo scenario, the bystander effect was observed in the rats fed with Ova and immunized with MBP + Complete Freund’s adjuvant (CFA) mixture. Ova was administered separately in the skin. These mice showed protection against the development of EAE. However, mice fed with Ova but immunized with MBP + CFA without separate Ova administration could not display any protection against the EAE development [49]. Thus, the antigen bystander effect appeared to be an essential phenomenon to induce peripheral tolerance against more than one antigen after the oral administration of a single antigen. At the preclinical level, bystander effect-mediated protection was operational in the streptococcal cell wall-induced arthritis after oral ingestion of collagen type II antigen [50].

## 4. Tools and Approaches for Antigen-Specific Oral Immune Tolerance

The success of oral immune tolerance depends on the nature, dosage, and frequency of the administered antigen, the innate background of the recipient, and the extent of antigen uptake by APCs of the gut-associated lymphoid tissue. In the harsh environment of the GI tract, the amount of antigen delivered to the intestinal site is minimal, probably due to the low bioavailability of the antigen at the target site because of possible degradation in the GI environment [51]. Therefore, rationalizing efficient delivery systems for the regulated release of antigens at the target site becomes essential. Several approaches were demonstrated to achieve oral immune tolerance, including natural and synthetic delivery systems.

### 4.1. Synthetic Nanoparticles

The particulate systems in nano and micron size ranges were explored to deliver different antigens to achieve peripheral immune tolerance. For instance, collagen type II (CII)-encapsulated poly(lactide-*co*-glycolide) (PLGA) nanoparticles (NPs) suppressed autoimmunity in collagen-induced arthritis (CIA), and synthesis of NPs was performed through the double-emulsion method. Mice administrated with a single dose of CII-containing PLGA particles reduced the clinical symptoms of experimental arthritis and systemic CII-specific antibodies [52]. In another study, NPs made of a polyethylene glycol (PEG) copolymer and PLGA-encapsulated insulin induced a hypoglycemic effect in type 1 diabetic mice [53]. A cationic charge-based polymer, polyethylene imine (PEI)-based NPs, were beneficial in diabetic rats through oral antigen delivery. NPs-based oral formulations successfully induced antidiabetic effects in diabetes rat models [54].

Metallic nanoparticles loaded with antigens/drugs are also helpful in inducing oral immunotherapy. For instance, the therapeutic efficacy of drug-loaded silver nanoparticles (Ag-NPs) was tested against autoimmune arthritis. Oral administration of Hesperidin-loaded lecithin-folic acid-based silver nanoparticles (Ag-NPs) after emulsification in CFA in an induced rat arthritis model led to anti-rheumatic activities. In addition, the oral treatment showed promising effects on relieving arthritis symptoms, such as the alleviation of joint inflammation, cartilage depletion, and bone erosion, by downregulating the secretion of anti-inflammatory cytokines (IL-6, IL-1β and TNF-α) while upregulating of the anti-inflammatory cytokine TGF-β.

Moreover, a low level of M1 macrophage activation and low production of RANKL, a receptor activator of the nuclear factor kappa beta ligand, and matrix metalloproteinase (MMP)-2/9 are associated with Ag-NPs-based oral immunotherapy [55]. In this series, drug-encapsulated lipid nanoparticles (L-NPs) in alleviating type 1 diabetes in a mouse model were helpful. Tofacitinib (Tofa)-encapsulated lipid nanoparticles exemplify this direction. L-NPs offer the advantages of negligible toxicity, good encapsulation efficiency, and a high level of phagocytosis by the immune cells. Moreover, upon oral administration, L-NPs uniquely accumulate in the lymphoid tissues, especially pancreatic and mesenteric lymph nodes. Interestingly, oral gavage of these Tofa-loaded L-NPs delayed type 1 diabetes onset in the NOD (non-obese diabetes) mice. NPs-based treatment is associated with localized inhibition of APC maturation while promoting T cell anergy [56].

### 4.2. Plant-Based Antigen Delivery

Upon ingestion, the transgenic plant-based systems expressing autoantigens, such as insulin and glutamic acid decarboxylase 65 (GAD), delivered intact proteins to the gut lymphoid tissue [57,58]. For instance, two autoantigens, human proinsulin fused with cholera toxin subunit B (CTB) and GAD, were expressed in lettuce and tobacco plants. Later, their efficacy was tested in non-obese diabetic (NOD) mice to suppress the insulitis condition [59]. CTB acts as a mucosal carrier to deliver the insulin antigen at the intestinal site to activate the immune system and correct aberrant autoimmune responses. Similarly, transgenic rice expressing three different peptide ligands and a T cell epitope of CII (CII256-271) protected against experimental arthritis development in DBA/1 mice. IL-10-dependent inhibitory mechanisms underlie the transgenic rice-based oral immunotherapy [60]. In another study, the *Arabidopsis thaliana* transgenic plant expressing a collagen-based fusion protein, CTA1(R7K)-COL-DD, prevented arthritis development in the same strain of mice with mild arthritic symptoms and a low CD4^+^T cell response, compared to the control group [61].

## 5. Antigen Nonspecific Oral Immune Tolerance

Apart from antigens, other therapeutic molecules, including hormones, antibodies, and natural products, have been demonstrated after oral administration to suppress or prevent autoimmune symptoms.

### 5.1. Therapeutics

Hormones, especially polypeptide hormones secreted from the pituitary gland, can control autoimmune responses. In a recent study, the therapeutic efficacy of adrenocorticotropic hormone (ACTH) was demonstrated after oral administration in a mouse model to alleviate the clinical symptoms of MS disease. Orally administrated ACTH at a specific frequency decreased the expression of IL-17-positive T cells in the gut lamina propria and spleen by activating the CD4^+^Foxp3^+^ Tregs in EAE mice [62]. A different mouse strain, SJL/J, was also treated with ACTH to verify the hormonal therapeutic effects. ACTH-treated mice showed improved MS clinical symptoms by downregulating the levels of IL-6, but with increased Treg cells in the lamina propria and a low population of CD4^+^ and γδ IL-17 cells in the central nervous system [63].

Commercially available monoclonal antibodies are promising in ameliorating disease symptoms after repeated oral feeding to the mice. For instance, the oral impact of tocilizumab (TCZ, Actemra^®^) was proven using C57BL/6 (B6) mice. TCZ-fed mice inhibited EAE onset and CNS inflammation by upregulating Th2 cytokines while concomitantly downregulating the Th1-type cytokines [64]. In a different study, the effect of Ustekinumab (Stelera^®^), a drug for psoriasis, was tested against EAE development in B6 mice. Mice orally fed with the UTZ drug suppressed the ongoing inflammatory responses.

Moreover, adoptively transferred splenocytes showed protection against EAE in mice immunized with myelin oligodendrocyte glycoprotein (MOG) peptide 35–55 by suppressing the synthesis of proinflammatory cytokines while increasing the production of Th2 cytokines [65]. Recently, the inhibition of Bruton’s tyrosine kinase (BTK) was demonstrated as a possible therapeutic option to control inflammation of autoimmune disorders by modulating the lymphocytes and innate immune cell signaling pathways. In a recent study, the efficacy of a BTK inhibitor, Evobrutinib, was tested in an autoimmune arthritis model by repeatedly orally feeding the rats. One daily dose of compound 36R for up to eleven days significantly reduced the diameter of ankle swelling in the arthritic joints compared to healthy ones. Moreover, no change in body weight was observed during oral administration of the drug, proving a good safety profile [66].

### 5.2. Probiotics

Recently, probiotics have shown promising results in suppressing or blocking autoimmune responses. For example, the therapeutic effects after oral administration of probiotics in the form of a mixture of *Bifidobacterium animalis* PTCC 1631 and *Lactobacillus plantarum* in B6 mice, with a daily dose, reduced the induction of EAE. In addition, mice fed with probiotics had a delay in disease onset and showed mild clinical signs. Moreover, a high percentage of Tregs was observed in the mouse spleen and lymph nodes, which received an oral probiotics treatment [67]. In a different study, probiotic and milk supplement effects were tested against MS. A probiotic *Bacillus amyloliquefaciens* supplement in camel milk fed to mice significantly reduced the disease symptoms by increasing the intestinal flora and short-chain fatty acids production in a MOG protein-induced EAE model in B6 mice.

Probiotic-fed mice had regulated levels of proinflammatory cytokines and myeloperoxidase [68]. Interestingly, some genetically engineered probiotic strains were far more effective in eliciting an immune tolerance. For instance, a genetically engineered *Lactobacillus reuteri*-based oral system was tested against rheumatoid arthritis. The *Lactobacillus reuteri* secretes the kv1.3 potassium blocker component Shk-235 (Lrs235) to control the ion channel activity, which blocks the proliferation of human T-effector memory lymphocytes. Interestingly, a single-time oral gavage of the engineered system significantly suppressed the inflammation in a delayed-type hypersensitivity reaction occurring in the atopic dermatitis animal model. 

Moreover, a daily oral dose of the engineered probiotic reduced the clinical symptoms of arthritis and joint inflammation in arthritis rats [69]. Another study tested the efficacy of a genetically modified strain, HSP-65, producing *Lactococcus lactis* in preventing and controlling EAE development in B6 mice. In both regimens, everyday oral administration of the HSP-producing bacterial strain reduced the MS-associated clinical symptoms by activating the LAP^+^CD4^+^Foxp3^+^ T cells in the spleen and the inguinal lymph nodes. Moreover, treated mice showed reduced leukocyte adherence in the venule structure of the spinal cord [70].

### 5.3. Plant-Based Products

Plant-based products have shown promising results in preclinical studies in achieving successful immune tolerance. For example, oral administration of the plant-derived “Baicalein” considerably reduced EAE clinical symptoms by blocking IL-17A production and suppressing MOG-specific antibodies. Moreover, Baicalein-based oral treatment reduced the pathogenic Th17 cells, CD4^+^-effector cells (CD44^hi^CD62L^low^), CXCR6^+^ CD4, CD8, and Th17 cells in the EAE mice [71]. In another study, the preventive effect of a natural alkaloid “Koumine” was demonstrated in the autoimmune hepatitis mouse model. This compound is one of the significant components of the traditional Chinese medicine, “Gelsemiumelegans”.

Concanavalin A injection was used to develop autoimmune hepatitis disease. In a dose-dependent manner, orally fed Koumine reduced the serum liver-associated inflammatory markers, such as alanine aminotransferase, aspartate aminotransferase, TNF-α, and IL-6, leading to the blocking of the development of hepatitis in mice. Mechanistically, Koumine regulates the expression of antioxidation factors such as Ho-1 and Nrf2 proteins by activating the Nrf-2 cell signaling pathway and altering the gut microbiota [72]. Different studies show the efficacy of a naturally derived component, “capsaicin,” in treating type 1 diabetes. Capsaicin acts as a ligand of vanilloid receptors expressed on the immune cells and neurons, thus regulating the immunological events in the gut. The oral ingestion of capsaicin in a specific dose attenuated the activation and proliferation of autoreactive T cells in the pancreatic lymph nodes. It protected the mice from developing autoimmunity. Capsaicin binding to vanilloid receptors enhanced CD11b^+^/F4/80^+^ immune cells in the pancreatic lymph nodes, which secreted anti-inflammatory proteins such as IL-10 and PD-L1 and controlled the inflammatory autoimmune responses [73].

## 6. Conclusions and Future Prospects

The oral route is a safe, robust, and more sustainable model to achieve tolerance by deleting the autoreactive T cells or generating T cell clonal anergy or Tregs. Though oral immune tolerance has shown promising results at the preclinical level, different challenges, including proper selection of antigen(s), dose optimization, and inappropriate immune system activation, hamper its progress to the next level. In the future, a more appropriate therapeutic target with fine-tuned dose optimization might be beneficial to achieve a successful immune tolerance strategy for ameliorating autoimmune disorders.

## Data Availability

Not applicable.
